# Psychological nursing intervention reduces psychological distress in patients with thyroid cancer: A randomized clinical trial protocol

**DOI:** 10.1097/MD.0000000000022346

**Published:** 2020-09-18

**Authors:** Lingling Wu, Yigang Zou

**Affiliations:** Department of Pain, Wuhan Fourth Hospital, Hubei, China.

**Keywords:** protocol, psychological nursing intervention, quality of life, thyroid cancer

## Abstract

**Background:**

Thyroidectomy has been considered an effective method to treat thyroid cancer. However, about 20% of patients have psychological distress before surgery. Psychological distress is considered common mental illnesses and it has been reported that the patients who suffer psychological distress have poor clinical outcomes than the patients without psychosocial disorder. Therefore, we design this randomized controlled study to explore the effect of psychological nursing intervention against quality of life and psychological distress of the patients with thyroid cancer.

**Method:**

The trial will be conducted from September 2020 to December 2020 at Wuhan Fourth Hospital on the basis of the International Council for Harmonisation's Good Clinical Practice Guidelines and the principles of the Helsinki Declaration. The study was authorized via the Research Ethics Committee of the Wuhan Fourth Hospital (Approval number: 20200721-046). This study is a single-center, randomized, 2-arm, evaluator-blinded clinical trial. In all, 90 patients with thyroid cancer undergoing thyroidectomy will be enrolled in this study. The inclusion criteria includes: patients aged between 20 and 60 years old; ASA I-II classification; normal platelet coagulation and count function. The exclusion criteria contains: people with the intellectual and cognitive impairment (behavioral-cognitive intervention); BMI above 35 kg/m^2^; the history of renal and hepatic dysfunction; and patients refuse to participate in this study. Both the patients in psychological intervention group and control group should receive the routine care, while the psychological intervention group also needs to receive the additional proper psychological nursing interventions. The emotional disorders are detected with the Chinese version of Profile of Mood States-Brief. And the patients’ life quality is evaluated with the European Organization for Research and Treatment of Cancer Quality of Life Questionnaire-Core Questionnaire (QLQ-C30, version 3.0). All the data are collated into Microsoft Excel 2010 and analyzed with SPSS 12.0 (IBM).

**Results:**

It is assumed that psychological nursing intervention could alleviate the psychological distress of patients with thyroid cancer and improve their quality of life.

**Conclusion:**

This study can provide the reliable evidence regarding the influence of psychological nursing intervention against the life quality and psychological distress of the patients with thyroid cancer.

**Trial registration:**

This study protocol is registered in Research Registry (researchregistry5937).

## 1 Introduction

Due to the rapid rise in incidence of thyroid cancer, it is now recognized as a major public health problem globally.^[1–3]^ It is the most familiar kind of endocrine malignancy and it causes more deaths than all other endocrine cancers combined. In the United States, from 1975 to 2009, the annual incidence increased from 14.9 per 100,000 to 14.3 per 100,000, almost tripling.^[4]^ Similarly, in the People's Republic of China, the incidence increased 2.4 times from 1988 to 2009, with 6.0% average annual growth rate.^[5]^ Differentiated thyroid carcinoma originates from the thyroid follicular cells and consists of follicular histological and papillary types, accounting for 90% of all the thyroid cancers.^[6]^

Thyroidectomy has been considered an effective method to treat thyroid cancer.^[7]^ However, about 20% of patients have psychological distress before surgery. Psychological distress is considered common mental illnesses and it has been reported that the patients who suffer psychological distress have poor clinical outcomes than the patients without psychosocial disorder.^[8]^ Even if we consider their importance, only few research has tried to manage the disorder. It is necessary to construct proper communication channels with the patients, establish positive harmonious relationship, and encourage cooperation, thus guiding patients to face the reality of the disease bravely.

Psychological nursing intervention is to carry out psychological nursing guidance for the patients, so that they can keep peace of mind, and then cooperate with the relevant treatment and nursing actively.^[9]^ Literature reports that in the clinical study, the effective psychological intervention can help to improve the patients’ psychological quality, and then enhance their life quality, which possesses great clinical significance.^[10]^ However, it is remained controversial regarding the effect of psychological nursing intervention in patients with thyroid cancer due to the small published articles. Therefore, we design the randomized controlled research to explore the effect of psychological nursing intervention against the life quality and psychological distress of the patients with thyroid cancer.

## 2 Methods

### 2.1 Study design

The trial will be conducted from September 2020 to December 2020 at Wuhan Fourth Hospital on the basis of International Council for Harmonisation's Good Clinical Practice Guidelines and the principles of the Helsinki Declaration. The research was authorized via the Research Ethics Committee of the Wuhan Fourth Hospital (Approval number: 20200721–046) and registered with in research registry (researchregistry5937). This study is a single-center, randomized, 2-arm, evaluator-blinded clinical trial. In all, 90 patients with thyroid cancer undergoing thyroidectomy will be enrolled in this study. The recruited patients are given the written informed consent before enrollment. The inclusion criteria contains: aged 20 to 60 years; ASA I-II classification; normal platelet coagulation and count function. The exclusion criteria contains: people with the intellectual and cognitive impairment (behavioral-cognitive intervention); BMI above 35 kg/m^2^; the history of renal and hepatic dysfunction; and patients refused to participate in this study.

### 2.2 Psychological nursing intervention

Both the patients in psychological intervention group and control group should receive the routine care, while the psychological intervention group also needs to receive the additional proper psychological nursing interventions. The contents of psychological nursing intervention are illustrated in Table 1.

**Table 1:**
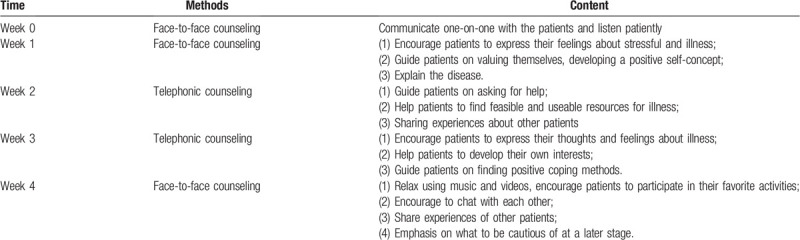
Contents of the psychological nursing intervention.

### 2.3 Psychological distress

The emotional disorders are detected with the Chinese version of Profile of Mood States-Brief.^[11]^ It involves 6 subscales (anger, fatigue, tension, depression, vigor, and confusion). Each subscale contains some adjectives (e.g., panic, unpleasant, sleepy, tired, and so on). There are 65 adjectives in total. And the items in each subscale are mixed. There are 5 levels for each answer: 0 represents no; 1 is a little; 2 represents media; 3 is a lot; and 4 represents very much. Total mood disorders = the negative mood score (sum of anger, depression, stress, confusion, and fatigue) minus the positive mood score (sum of self-esteem and vitality) and then plus the constant (100). A higher score corresponds to a more negative emotional state. The anxiety and depression are measured through utilizing the Hospital Anxiety and Depression Scale. The table consists of 2 subscales of depression and anxiety, each of which involves 7 questions about depression and anxiety. Each question has 0 to 3 points and 4 answers. The scores of each subscale of anxiety and depression are 0–7+ that represents asymptomatic; 8–10+ points that is suspicious; and 11–21+ points that represents definitely existed; when scoring, the starting point is 8 points. Both symptomatic and suspicious are positive.

### 2.4 Quality of life

The patients’ life quality is evaluated with the European Organization for Research and Treatment of Cancer Quality of Life Questionnaire-Core Questionnaire (QLQ-C30, version 3.0).^[12]^ It is divided into 3 symptom fields (pain, fatigue, nausea, vomiting), 5 functional fields (cognitive function, role function, physiological function, social function, and emotional function), 1 area of quality of life/entire health status, as well as 6 separate items (each as a field). The scoring rules explicitly state that higher scores for the overall health conditions and functional areas are associated with better quality of life and functional status.

### 2.5 Statistical analysis

SPSS Sample Power 3.0 software (IBM, Armonk, NY) is used to calculate the sample size. For the purpose of this study, the baseline data of outcome variables are utilized as covariate, and the needed sample size is estimated to be 45 participants in each group via independent-samples one-way analysis of covariance (ANCOVA) to detect the effects of power of 0.8 and alpha of 0.05. All the data are collated into Microsoft Excel 2010 and analyzed with SPSS 12.0 (IBM). The comparison of baseline characteristics of the patients between these 2 groups is carried out through utilizing the *χ*^2^ test for categorical data or the independent-sample *t* tests for continuous data. Intention-to-treat analysis is used for the outcome assessments. The linear mixed patterns are utilized to assess the difference of life quality and psychological distress. The *P* < .05 is regarded as statistically significant.

## 3 Results

It is assumed that psychological nursing intervention could alleviate the psychological distress of patients with thyroid cancer and improve their quality of life.

## 4 Discussion

Nowadays, it is extensively believed that the psychological factors play an important role in the process of canceration, meaning that the cancer patients may be more likely to report psychological symptoms such as increased stress, depression and anxiety, etc. unconsciously or consciously than the patients with benign tumors.^[13,14]^ In the modern medicine, education in health has become a significant part of treatment. For example, psychological care is utilized to intervene in the lives of patients with mental health problems, thereby improving the treatment compliance. More and more studies have emphasized the importance of psychological nursing intervention in reducing psychological distress in patients with cancer.^[15,16]^ At the same time, the nurses should try to reduce any adverse stimulation, eliminate harmful psychological barriers, and analyze the emotional changes and thinking process of patients, encourage the patients to express their true feelings, so that they can express their negative emotions safely.^[17,18]^ Our current work is the first clinical randomized controlled trial to explore the effect of psychological nursing intervention against quality of life and psychological distress of the thyroid cancer patients. These findings must be replicated in more research centers and in larger samples, and evaluated over longer periods of follow-up, before final conclusions can be drawn.

## 5 Conclusion

This study can provide the reliable evidence regarding the influence of psychological nursing intervention against the life quality and psychological distress of the patients with thyroid cancer.

## Author contributions

Yigang Zou designed and reviewed protocol. Lingling Wu performed the data collection and analysis. Lingling Wu finished the manuscript. All of the authors approved the submission.

**Conceptualization:** Yigang Zou.

**Funding acquisition:** Yigang Zou.

**Writing – original draft:** Lingling Wu.

## Abbreviations

Abbreviation: QCQ = questionnaire-core questionnaire.
